# Pediatric suicide by violent means: a cry for help and a call for action

**DOI:** 10.1186/s40621-022-00378-6

**Published:** 2022-04-08

**Authors:** Christina M. Theodorou, Kaeli J. Yamashiro, Sarah C. Stokes, Edgardo S. Salcedo, Shinjiro Hirose, Alana L. Beres

**Affiliations:** 1 Division of Pediatric General, Thoracic, and Fetal Surgery, Department of Surgery, University of California Davis Medical Center, 2335 Stockton Blvd, Room 5107, Sacramento, CA 95817 USA; 2 Division of Trauma, Acute Care Surgery, and Surgical Critical Care, Department of Surgery, University of California Davis Medical Center, 2335 Stockton Blvd, Sacramento, CA USA

**Keywords:** Pediatric suicide, Pediatric trauma, Firearm safety, Psychiatric illness

## Abstract

**Background:**

Suicide is the second most common cause of death among adolescents and young adults. In the pediatric population, gunshot wounds (GSWs) and hangings are common mechanisms of pediatric suicide. Comorbid psychiatric illness is prevalent in this population, but psychiatric resource utilization after self-inflicted traumatic injury is not well characterized.

**Methods:**

We analyzed patients < 18 years old presenting to a level 1 pediatric trauma center after suicide attempt by GSW, hanging, or jumping from a height from 2009 to 2019. The primary outcome was psychiatric resource utilization. Secondary outcomes included prior emergency department (ED) visits to identify prior opportunities for intervention.

**Results:**

Of 6538 pediatric trauma patients, there were 219 GSWs, 7 hangings, and 7 jumps from height, for a total of 233 patients. Of these, 14 presented following a suicide attempt (four GSWs, six hangings, and four jumps, total 6.0%). Half of these patients died due to their injuries. Self-inflicted GSWs had the highest mortality (75%). Most surviving patients were placed on involuntary psychiatric holds (*n* = 5/7, 71.4%), and three patients were discharged to an inpatient psychiatric hospital (*n* = 3/7, 42.9%). Five of the 14 patients had prior ED visits (35.7%), and of these, 60% were for suicidal ideation or suicide attempts.

**Conclusions:**

Among pediatric trauma patients, suicide attempts are rare, but are highly lethal, with the highest mortality rate seen in self-inflicted GSWs. Psychiatric resource utilization is high both during and after the hospitalization. Prior ED visits may represent opportunities for depression and suicidality screening in this at-risk population.

## Background

Suicide is the second most common cause of death for children and young adults aged 10–34 years old in the USA (Centers for Disease Control and Prevention 2018). In children and adolescents, most suicides are by violent means, frequently gunshot wounds or hangings, accounting for 37% and 50% of pediatric suicides, respectively (Trigylidas et al. 2016). This is supported by data from the Centers for Disease Control and Prevention (CDC) Wide-ranging OnLine Data for Epidemiologic Research (WONDER) database, which lists hanging and self-inflicted firearm injuries as the two most common self-inflicted mechanisms of injury resulting in death (https://wonder.cdc.gov/ucd-icd10.html). Self-inflicted firearm injuries have the highest mortality (Swendiman et al. 2020). Additionally, there are discrepancies between institutional case fatality rates among pediatric victims of firearm violence and epidemiologic data which has the benefit of including on-scene fatalities; this discrepancy highlights a hidden mortality of pediatric firearm violence (Theodorou et al. 2022). In the state of California alone, according to the California Department of Public Health Overall Injury Surveillance Tool (https://epicenter.cdph.ca.gov), from 2009 to 2015, there were 65,000 pediatric patients injured in non-fatal suicide attempts, accounting for 100 per 100,000 children and adolescents. Fatal suicides occurred much less frequently, at 1 per 100,000 children and adolescents, but were responsible for 634 deaths over the time period. These were most commonly by hanging (351 deaths) or by firearm (166 deaths). The magnitude of the problem is enormous.

Preventative efforts have focused on gun safety education for caregivers (Grossman et al. 2005) and depression and suicidality screening in primary care settings (Sisler et al. 2020). Access to firearms is a known risk factor for pediatric suicide (Flaherty and Klig 2020), and limiting access has been a focus of preventative measures (Mann and Michel 2016). Children and adolescents who attempt suicide or die by suicide have a high incidence of psychiatric illness, and most suicide attempts that result in the patient’s death are precipitated by an identifiable trigger in the patient’s life, most commonly interpersonal problems with a significant other (31%), with a parent (13%), or recent death of a loved one (8%) (Molina and Farley 2019). Outside of self-inflicted firearm injuries, there is a paucity of literature reporting on the epidemiology of patients presenting as trauma activations following suicide attempts (Miller et al. 2002; Li et al. 1997; McLoughlin et al. 2019). An understanding of the patients who attempt suicide by violent means, and the resources utilized, is critical for the pediatric providers caring for these patients.

We aimed to characterize the frequency of suicide attempts in pediatric victims of traumatic injury with identifiable self-inflicted mechanisms of injury who survived to present to the hospital, focusing on injury mechanisms with high rates of intentional self-harm, including firearm injuries, hangings, and jumps from height. We investigated rates of psychiatric resource utilization and prior presentations to the emergency department.

## Methods

### Study design and setting

Following institutional review board approval, our level 1 pediatric trauma center registry was queried for all pediatric traumas < 18 years old who sustained gunshot wounds (GSW), hangings, and jumps from height 1/1/2009–7/1/2019. Chart review was performed to determine if the injury was intentionally self-inflicted (suicide attempt). Patients were excluded if the injury was accidental, inflicted by another person, or if the injury intent could not be determined. The primary outcome was psychiatric resource utilization, defined as inpatient psychiatric consultation and discharge to acute inpatient psychiatric facilities following the index trauma hospitalization. Information was collected on demographics, injury mechanism, prior emergency department (ED) visits or trauma activations, prior psychiatric diagnoses, injury severity scores (ISS), hospital length of stay (LOS), and 30-day and 1-year ED visits and readmissions. Case fatality rates were calculated by mechanism.

Descriptive statistics were performed. Categorical data are presented as proportions and percentages. All continuous data were nonparametric and are presented as median and interquartile range (IQR). Analysis was conducted using SAS (SAS, version 9.45; SAS Institute Inc).

## Results

### Overall patient characteristics

From 1/1/2009 to 7/1/2019, there were 6538 pediatric trauma admissions. Of these, there were 219 GSWs, 7 hangings, and 7 jumps from height, for a total of 233 patients. One patient with a self-inflicted GSW to the head was excluded due to inability to determine if the injury was intentional. There were 218 patients with injuries inflicted by others (*n* = 199) or unintentional self-inflicted injuries (*n* = 19), including 215 patients injures by GSW, 1 accidental hanging, and 3 jumps from height without suicidal intent. Thus, fourteen patients were identified as presenting after intentional self-inflicted injuries (suicide attempts, 6.0%). The remaining trauma admissions were due to other mechanisms of injury, such as motor vehicle collisions, ground-level falls, or recreational injuries.

The fourteen patients who presented following suicide attempts were mostly male (*n* = 9/14), and adolescents (median age 16, range 8–17 years old) (Table 1). The mechanisms of injury were GSW (*n* = 4), hanging (*n* = 6), and jumps from a height (*n* = 4). Of the GSWs, three were to the head and the remaining patient sustained a GSW to the chest.

**Table 1:** Characteristics and outcomes of patients presenting following suicide attempt

	Suicide attempt *n* = 14
Age, years: median (IQR)	16 (13–17)
Sex, % male: *n* (%)	9 (64.3)
Transfer: *n* (%)	6 (42.9)
Weekday presentation: *n* (%)	7 (50.0)
Overnight presentation: *n *(%)	8 (57.1)
Mechanism: *n* (%)	
GSW	4 (28.6)
Jump	4 (28.6)
Hanging	6 (42.9)
ISS: median (IQR)	20.5 (4–25)
Hospital LOS, days: median (IQR)	2 (1–11)
ICU admission: *n* (%)	9 (64.3)
ICU LOS, days: median (IQR)	1.5 (1–6.5)
Mechanical ventilation: *n* (%)	8 (57.1)
Ventilator days: median (IQR)	1 (1–2)
Underwent surgical intervention: *n* (%)	3 (21.4)
In-hospital mortality: *n* (%)	7 (50.0)
Previous ED visits: *n* (%)	5 (35.7)
Previous trauma activations: *n* (%)	0 (0)

ED, Emergency department; GSW, gunshot wound; ICU, intensive care unit; IQR, interquartile range; ISS, injury severity score; and LOS, length of stay

### Outcomes

The in-hospital mortality rate was 50% (*n* = 7/14); two patients died in the ED and five died in the ICU. The case fatality rate was highest for self-inflicted GSWs (*n* = 3/4, 75%), followed by hangings (*n* = 4/6, 66.7%), with no deaths due to intentional jumps.

Seven patients survived, and all were seen by the inpatient psychiatry team (Table 2). Most were placed on involuntary psychiatric holds (*n* = 5/7, 71.4%), and three patients were discharged to an inpatient psychiatric hospital (*n* = 3/7, 42.9%). The four patients who were not discharged to an inpatient psychiatric hospital were cleared for safe discharge home with family by the psychiatry team. One patient returned to the ED within 30 days for pain related to their injury; there were no readmissions within 30 days. Over the course of 1 year following the index hospitalization, two additional patients returned to the ED, both for psychiatric concerns. In one case, the patient needed medication refills for their medications for bipolar disorder; in the other case, the patient was brought to the ED for acute psychosis and required an additional inpatient psychiatric hospitalization. Of the 7 surviving patients, outpatient psychiatric follow-up was documented for two patients within our electronic medical record system (28.6%).

**Table 2:** Outcomes for surviving patients

	Suicide attempt survivors *n* = 7
Inpatient psychiatry consult: *n* (%)	7 (100.0)
Involuntary psychiatric hold: *n* (%)	5 (71.4)
Discharge to psychiatric facility: *n* (%)	3 (42.9)
30-day ED visit: *n* (%)	1 (14.3)
30-day readmission: *n* (%)	0 (0)
1-year ED visit: *n* (%)	3 (42.9)
1-year readmission: *n* (%)	1 (14.3)
Documented outpatient psychiatry follow-up visits	2 (28.6)

ED, Emergency department

### Prior encounters

Five of the 14 patients had prior ED visits for any reason in our system (*n* = 5/14, 35.7%). One patient was seen 14 months earlier with suicidal ideation, and one patient was admitted for an involuntary psychiatric hold at a local institution 5 months prior. Two patients had ED visits for minor injuries. One patient had prior ED visits, but detailed records of those visits were unavailable. However, chart review of the hospitalization included in this study revealed the patient had been recently discharged from a prolonged psychiatric hospitalization for a suicide attempt. Thus, three of five patients (60%) who had been to the ED prior were for reasons related to suicidal ideation or suicide attempts. Overall, however, this cohort represented only 3 of the 14 total patients overall, (*n* = 3/14, 21.4%). There were no prior trauma activations.

Only six patients had a reported prior diagnosis of psychiatric illness (*n* = 6/14, 42.9%), including depression (*n* = 4), bipolar disorder (*n* = 1), schizophreniform disorder (*n* = 1), adjustment disorder (*n* = 1), post-traumatic stress disorder (*n* = 1), attention-deficit/hyperactivity disorder (*n* = 1), and not specified (*n* = 1). Most patients had multiple diagnoses. Five patients had no history of psychiatric diagnosis. For three patients, this information was not available; these three patients all died from their injuries.

## Discussion

In this retrospective review of pediatric and adolescent patients presenting as traumas following GSWs, jumps, or hangings, 6.0% of patients were injured in an attempt to end their own lives. Half of these patients died. All surviving patients received inpatient psychiatric consultations, and almost half were discharged to inpatient psychiatric facilities. One-third of patients had been seen in the ED prior, with three patients presenting for prior suicidal ideation or attempt (3/14, 21.4%). These prior ED visits represent a window of opportunity for screening for depression and suicidality, and for potential prevention of a future psychiatric health crisis that culminates in a suicide attempt.

Rates of suicide in children and adolescents have risen by 33% since 1999 (Hedegaard et al. 2018), and suicide is now the second leading cause of death from 10 to 34 years old (Centers for Disease Control and Prevention 2018). In a survey of high school seniors, up to 17% reported serious suicidal ideation in the preceding 12 months (Kann et al. 2018). The most common methods of suicide attempt resulting in death in children and adolescents involve asphyxiation, such as by hanging, and self-inflicted firearm injuries (Shain 2016), as seen in this study. Unfortunately, children and adolescents who attempt suicide by firearm frequently succumb to their injuries, with a reported lethality of up to 90% (Elnour and Harrison 2008). These findings are supported by our cohort, with the highest lethality for self-inflicted firearm injuries.

Psychiatric disorders are known risk factors for self-inflicted firearm injury requiring hospitalization in adolescents (McLoughlin et al. 2019). However, up to 54% of patients who die by suicide have no preexisting psychiatric diagnosis (Stone et al. 2018). This finding is supported by our study of this subpopulation of pediatric trauma patients, with less than half (42.9%) having a prior psychiatric diagnosis.

We found that 36% of patients who presented following suicide attempt had a prior ED presentation, and of these 60% had been previously treated for suicidal ideation or after a suicide attempt. In a statewide study of predictors of self-harm ED visits among adolescents, 57% of patients had prior ED visits, compared to 50% of patients presenting for other reasons. However, only 9% of patients who presented for self-harm had prior ED visits for self-harm, indicating that the majority of ED presentations prior to suicide attempts are for unrelated concerns (Goldman-Mellor et al. 2019). This is supported by a single-center study of 42 adolescent suicides, which found that patients infrequently had prior suicide attempts, in particular among those who sustained self-inflicted GSWs (Lyons et al. 2021). Routine screening of adolescents presenting to the ED for non-psychiatric concerns uncovered a 30% rate of depression, with concurrent suicidality in 5–10% of patients (Esposito et al. 2020). ED visits can be used as an opportunity to screen for depression and self-harm risk factors and may allow for earlier identification of at-risk youth and referral to appropriate services.

Critically important in preventing child and adolescent suicide is firearm safety. States with stricter child firearm access prevention laws have lower rates of adolescent suicide (Webster et al. 2004). Firearms were present in nearly half of homes with children who have risk factors for self-harm or suicide, and of these, more than one-third do not practice safe firearm storage (Scott et al. 2018). As access to firearms within the home is a known risk factor for pediatric suicide (Flaherty and Klig 2020), education on safe firearm storage is critical in preventing both intentional and unintentional access to firearms. Firearm safety screening in the ED, combined with routine depression or suicidality screening regardless of chief complaint, has the potential to reduce the morbidity and mortality associated with pediatric suicide.

Our study has several limitations. The sample size is small, but as patients were drawn from prospectively collected institutional database at a large level 1 pediatric trauma center, the overall low rate of pediatric suicide attempts is likely similar in other major trauma centers. Although database studies may provide higher numbers of patients, details regarding psychiatric care and follow-up are not available, limiting their utility to address these pressing questions. As such, this study is a descriptive study, and the ability to perform meaningful statistical analyses is limited. In this study, we attempted to identify the rate of outpatient psychiatric follow-up in this population, but were limited by the nature of the electronic medical record (EMR), and we unable to determine if patients who did not have outpatient psychiatry visits truly did not have follow-up, or if they had follow-up within a different EMR system. We additionally did not capture any patients that were declared dead at the scene, which may represent a significant proportion of pediatric suicides by violent means given their high lethality. This study can be used as a catalyst for future multicenter studies aiming to further evaluate predictors of violent suicide attempts among adolescents, and appropriate psychiatric needs during and after their hospitalizations.

## Conclusion

In conclusion, 6% percent of children injured by firearms, hanging, or jumps from height were the result of intentional acts of self-harm. These suicide attempts are highly lethal, with 50% of patients dying. Suicide by gunshot wound had the highest mortality. Psychiatric resource utilization in the inpatient setting and after discharge was high for surviving patients. Prior interaction with the healthcare system represents a window of opportunity for screening for depression, suicidality, and home safety and has the potential to save lives.

## Data Availability

The datasets analyzed during the current study are not publicly available due to patient confidentiality but are available from the corresponding author upon reasonable request.

## Abbreviations

GSW: Gunshot wound
ED: Emergency department
ISS: Injury severity score
LOS: Length of stay
IQR: Interquartile range
ICU: Intensive care unit
